# Supplementary material for: Signatures of the sub-Rayleigh to supershear fracture transition in snow avalanche experiments
Source: Nat Commun. 2025 Dec 16;16:11153. doi: 10.1038/s41467-025-65825-6 (PMC12708799; doi:10.1038/s41467-025-65825-6)
Supplement: Supplementary file 1 — Supplementary Information [file 41467_2025_65825_MOESM1_ESM.pdf]

Supplementary Information for

Signatures of the sub-Rayleigh to supershear fracture transition in snow avalanche experiments

Bergfeld et al.

To whom correspondence should be addressed; E-mail: johan.gaume@slf.ch

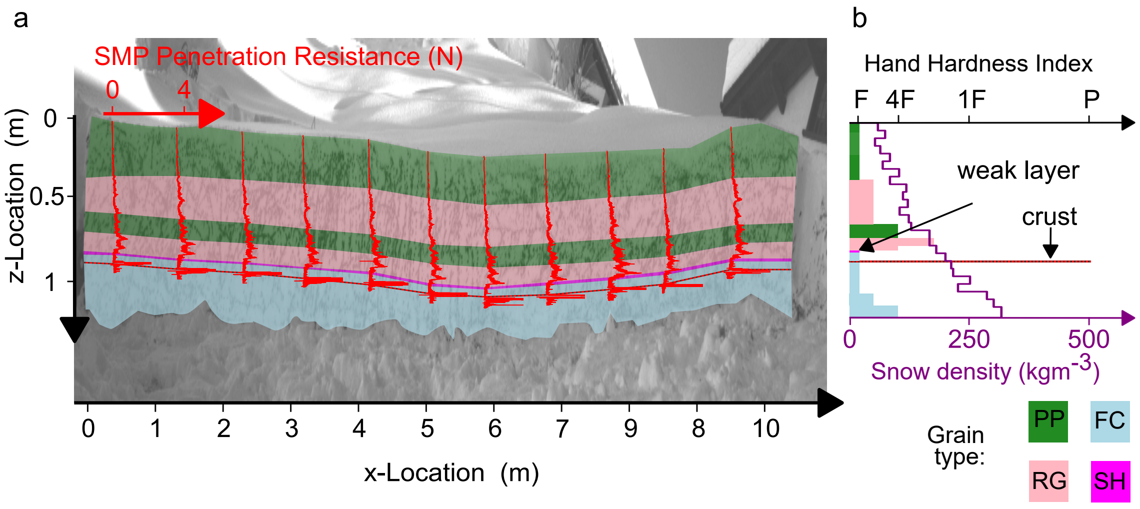

Supplementary Figure 1: **a**, SMP-measured penetration resistance (red lines plotted on the PST image) measured between PST #2 and PST #3. The coloring of the background corresponds to the layers identified in the manual profile (**b**). The manual profile shows the hand hardness index. Grain shape is indicated by colors and two two-letter code (1). The slab consisted mainly of precipitation particles (PP) and rounded grains (RG), the weak layer was a layer of surface hoar (SH) and the substratum consisted of faceted crystals (FC). The snowpack had increasing density with depth (purple line in the profile).

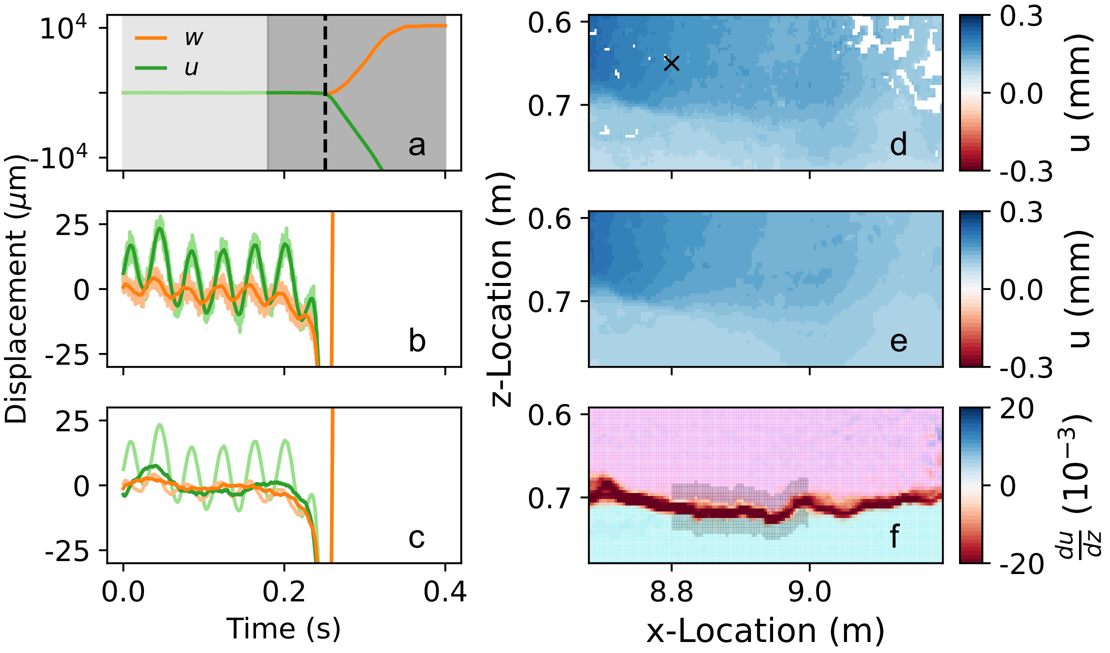

Supplementary Figure 2: **a**, Digital Image Correlation (DIC) of video recordings provides slope-normal displacement (w, orange) and the tangential displacement (u, green) of an individual subset (cross in **d**) with time. Initially, the subset is at rest (light, gray background). Subsequently, the crack propagates through the close-up, causing the slab to displace towards the substratum (orange) and causing the slab to slide downslope (green, dark grey background). The raw displacement signal contains two kinds of noise. **b**, High frequency noise (transparent green and orange) which was filtered with a Savietzky-Golay filter. **c**, Secondly, a superposition of the displacement with a sine wave ($\approx 25\,\mathrm{Hz}$) is noticeable. It is caused by the rotation of the camera fan and is more pronounced for the tangential displacement (**c**, light green). A sine wave was fitted to the signal and subtracted (green, orange) to eliminate this effect. At the time (**a**, dashed line) the crack propagates through the field of view, tangential displacement in the slab is increasing from right to left. **d**, there are still missing measuring points (white area) and displacement fields have not been spatially smoothed. **e**, Voids have been in-painted using a contextual-recovery algorithm (2; 3) and a three dimensional Non-Local-Mean filter was applied (4). **f**, Based on the strain field shortly after the crack propagated through the FOV ($5\,\mathrm{ms}$, not highlighted in **a** because the time difference cannot be distinguished from the dashed line at this scale) subsequent to the fields (**d**) and (**e**), the slab (transparent pink) and substratum (transparent turquoise) were identified as the regions above and below the strain localization across the weak layer. The centered grayish regions around the weak layer were used to observe differences in displacement between the crack faces.

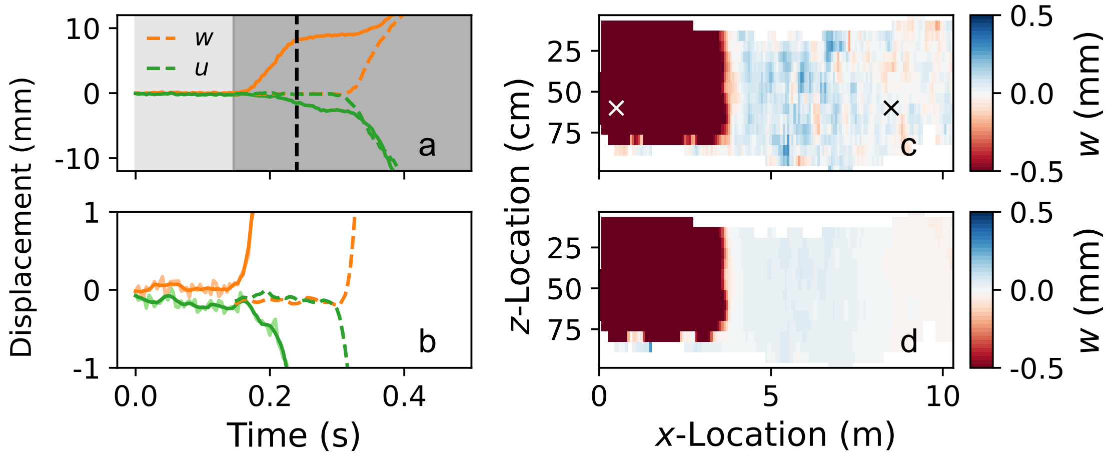

Supplementary Figure 3: Pre-processing of full-view recordings. **a**, Digital Image Correlation (DIC) of video recordings provides slope-normal displacement (w, orange) and the tangential displacement (u, green) of individual subsets (crosses in **c**) with time. Initially, the subset is at rest (light, gray background). Subsequently, a crack propagates (dark gray background), causing the slab to displace towards the substratum (orange) and causing the slab to slide downslope (green). Subsets close to the sawing end of the Propagation Saw Test (white cross in **c**, solid lines in **a** and **b**) displace earlier in time than a subset towards the far end of the test (black cross in **c**, dashed lines in **a** and **b**). **b**, Raw displacement signals contain noise (transparent green and orange) which was filtered with a Savietzky-Golay filter (solid and dashed lines). **c**, Slope-normal displacement w, of the PST side wall at a time shown in **a** with the dashed black line. The increase in displacement indicates the approximate position of the crack tip. In **d**, visible noise ahead of the crack tip (larger x-locations, **c**) was reduced using a three-dimensional NLM filter (4).

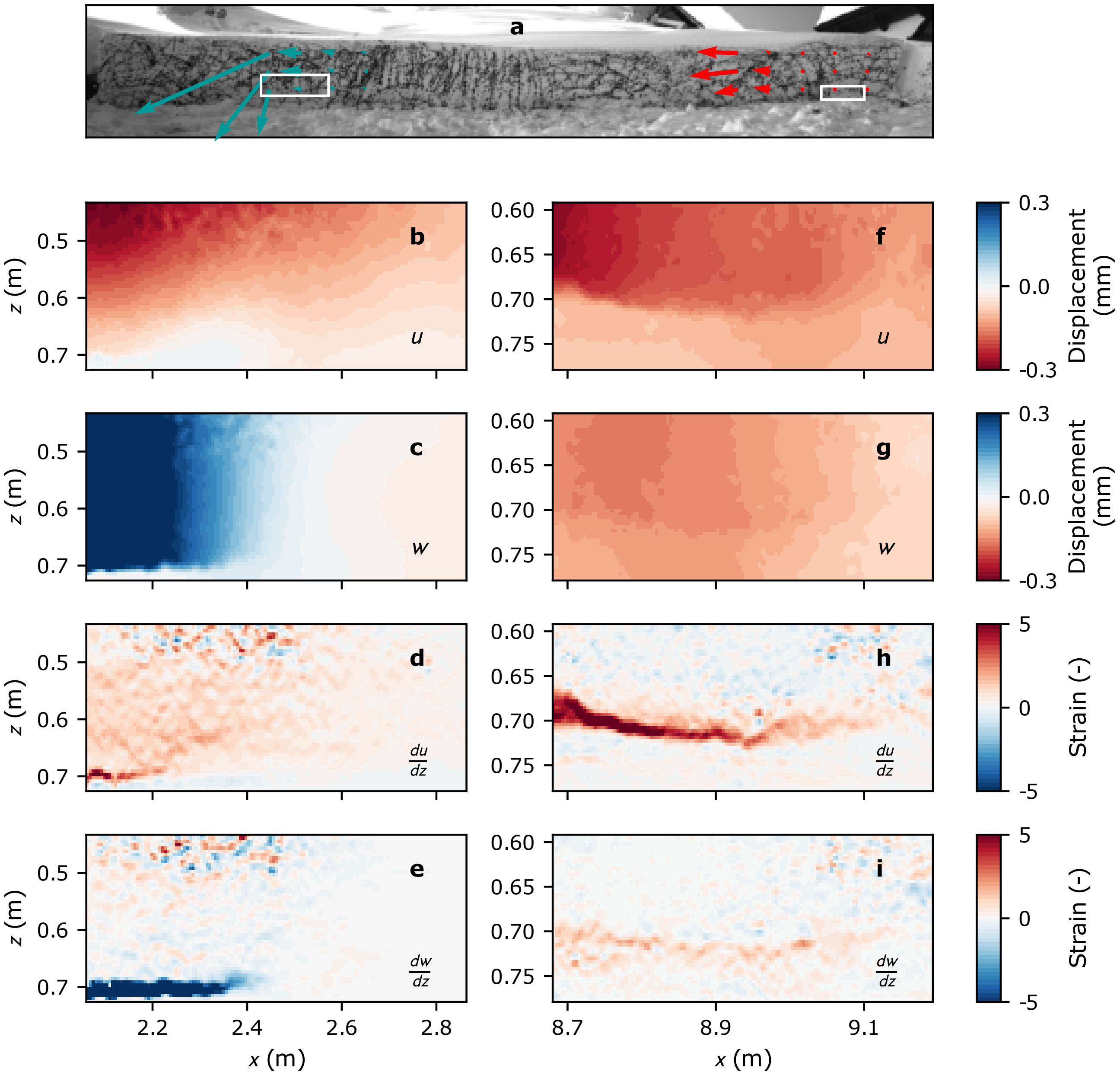

Supplementary Figure 4: Deformation analysis. **a**, Spatial patterns of displacement vectors. Tangential displacement (**b**), normal displacement (**c**), shear strain (**d**) and normal strain (**e**) at close-up #1. Tangential displacement (**f**), normal displacement (**g**), shear strain (**h**) and normal strain (**i**) at close-up #2. The plots are shown for two time instances corresponding to the passage of the crack tip at the locations of close-up #1 ($\Delta t^{cu1} = 14.3$ ms) and close-up #2 ($\Delta t^{cu2} = 21.8$ ms), respectively. Hence, the same times that were used in Figure 3.

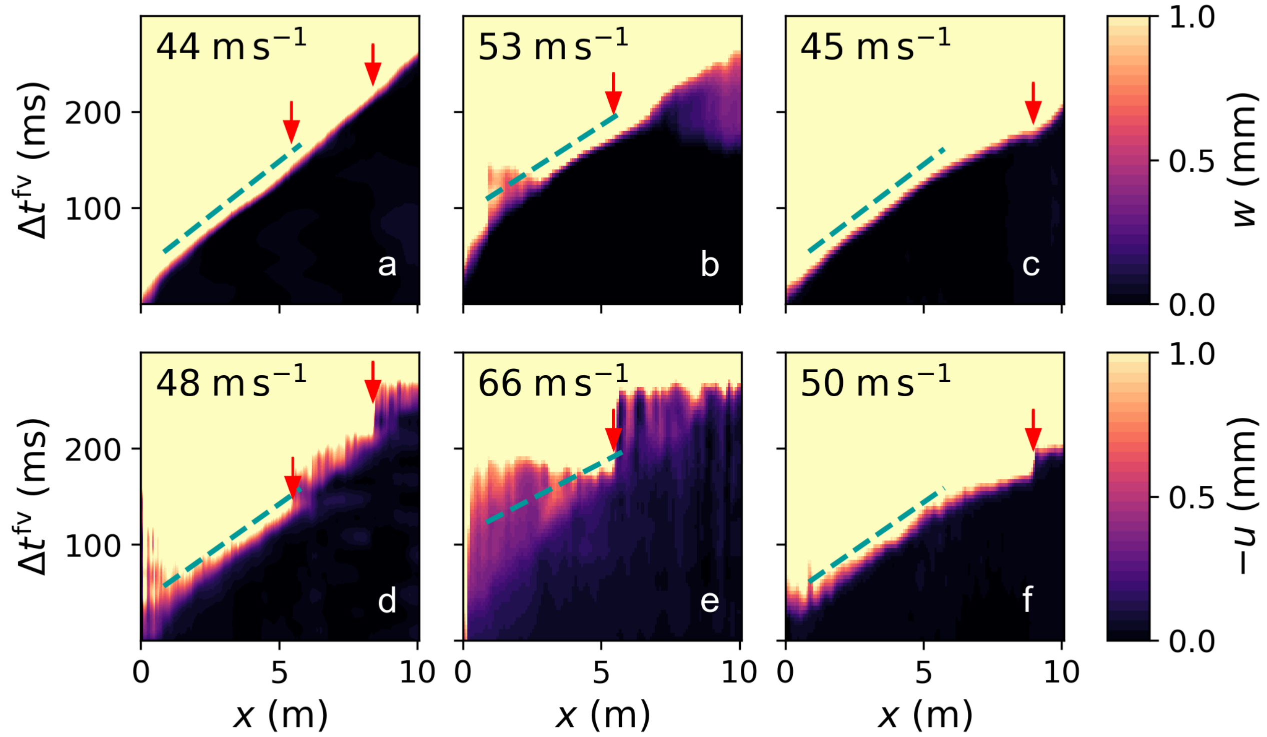

Supplementary Figure 5: Slope-normal displacement $w(t)$ with time for the three PST experiments (**a**,**b**,**c**). The slope-parallel displacement $u(t)$ is shown at the bottom (**e**,**f**,**g**). Slab fractures are shown with red arrows pointing at x-location where they appeared.

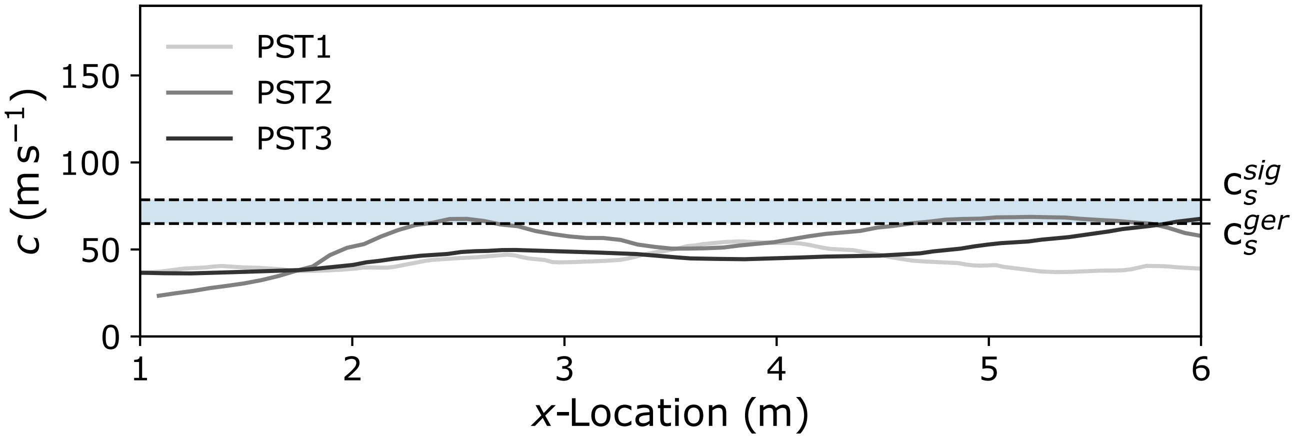

Supplementary Figure 6: Crack propagation speed obtained on the basis of the slope-normal displacement $w(t)$ (same threshold of $0.467\,\mathrm{mm}$ was used for speed estimation) in the three PSTs in the first 5 m of crack propagation. In this range where no PST had a slab fracture, slab deformation as well as measured crack speeds in all three PSTs were similar and revealed mixed-mode anticrack propagation.

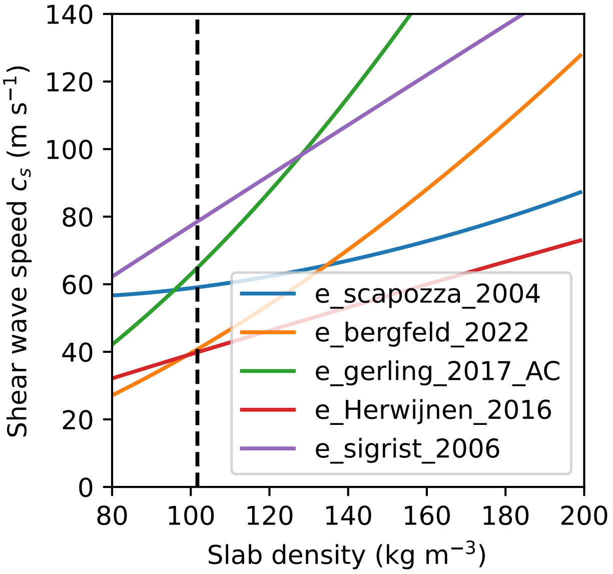

Supplementary Figure 7: Shear wave velocity with snow density. The dashed line represents the mean slab density of the experiments. Depending on the parametrization (5; 6; 7; 8; 9) used to compute an elastic modulus from density, shear wave speeds range from 40 to 79 ms^{-1}.

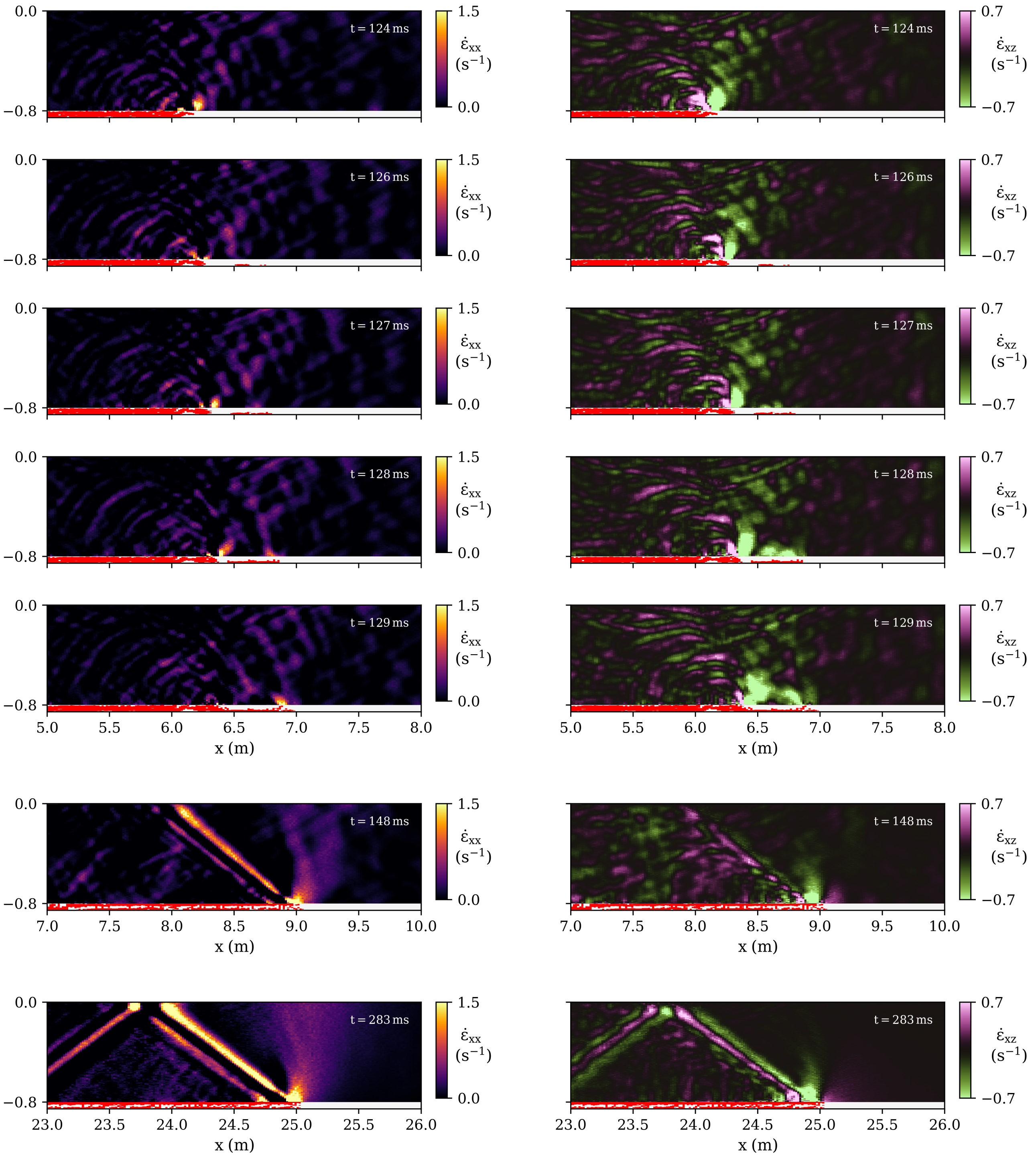

Supplementary Figure 8: Evidence of the Burridge Andrews mechanism in MPM simulation of the PST #3 experiment. Plasticized particles in the weak layer are colored in red and the slab is colored by the longitudinal rate of deformation (left) and shear rate of deformation (right). The supershear transition occurs at approximately 5.5 m, consistent with the transition reported in PST #3. A Mach cone begins to form beyond this point, becoming distinctly visible once a steady state is achieved.

Supplementary Table 1: Camera and digital image correlation (DIC) settings. The DIC subsets were allowed to translate, rotate and deform with normal and shear. For the subset initialization, interpolation and optimization method we used the *feature matching*, *keys fourth* and *gradient based* setting of the DICe (Digital Image Correlation Engine) software.

Perspective	full-view			close-up	
PST #	1	2	3	2	3
Camera	HS	Sony	Sony	HS	HS
	camera settings				
Resolution (pixel^2)	1280×200	1920×1080	1920×1080	1280×504	1280×504
Frame rate (s^{-1})	5000	250	250	10000	10000
Exposure time (μs)	199.6	–	–	99.0	99.0
Focal length (mm)	24	–	–	70	70
Aperture (-)	2.8	–	–	2.8	2.8
	DICe settings				
Subset size (pixel)	11	29	29	27	27
Step size (s^{-1})	4	9	9	9	9
Number of subsets (-)	7300	1529	1998	6784	6641
Threshold (-)	40	24	24	40	40
Pixel conversion[1] (mm pixel^{-1})	8.8	8.9	7.0	0.6	0.4
	Displacement uncertainty				
DIC uncertainty (mm)	0.6	0.04	0.018	0.010	0.0011
Standard deviation of displacements (mm)	0.4	0.06	0.4	0.005	0.004

[a]Truncated after the first decimal digit.

Supplementary Table 2: MPM simulation parameters for Figure 5 and Supplementary Fig. 8

MPM Simulation Parameters		Slab	Weak Layer
Geometry	Thickness (m)	0.8	0.05
	Length (m)	30	
	Slope angle (°)	37	
Material properties	Poisson's ratio ν	0.2	
	Density ρ (kg m^{-3})	102	110
	Young's modulus E (MPa)	1.52*	0.7
	Initial consolidation pressure p_0 (kPa)	-	23
	Tension/compression ratio β	-	0.3
	Friction coefficient M	-	1.0
	Hardening factor ξ	-	0.008
Numerical model	Element size dx (cm)	0.7	
	Mean number of particles per cell	4	
	CFL	0.4	
	Frame rate (s^{-1})	1000	

* Derived from Sigrist's formula $E = 1.89\rho^{2.94}$

Supplementary Note 1 – Slab fractures

Slab fractures (SFs) are a known phenomenon that may prevent crack propagation, or influence its dynamics. We observed one or more slab fractures in our PSTs. In general, SFs occur where the tensile stress within the slab exceeds the tensile strength (10). The strength is primarily determined by slab density, whereas the tensile stress is a superposition of the downslope tension of the slab and the tensile stress caused by slab bending. As the downslope tensile stress in the slab increases with crack propagation distance, the slab will always fracture after a certain propagation distance. The respective effect of a slab fracture depends on its relative position to the crack tip in the weak layer. During the sawing phase of a PST, i.e. before the onset of crack propagation at a very short crack length, the gravitational pull is negligible and the tensile stress originates mainly from bending. If the slab cannot withstand this deformation, no unstable crack growth is possible. However, the slab can typically withstand the bending stresses up to the onset of crack propagation. If so, an anticrack starts propagating and the increasingly larger detached part of the slab increases gravitational downslope pull and therefore tensile stresses in the slab. If the slab resists the tensile stresses up to the super critical crack length, crack propagation transitions into supershear propagation regime (11).

In PST #1 and PST #2 we observed SFs at around $x = 5.5\,\mathrm{m}$. That is approximately at the super-critical crack length observed in PST #3. In PST #3, the absence of the slab fracture at $x = 5.5\,\mathrm{m}$ was speculatively due to the progressive natural strengthening of the slab induced by densification (12), which is rapid for low density snow consisting of precipitation particles (13). A transition into the supershear crack propagation regime was not observed in the two earlier experiments (PST #1, PST #2). In PST #3, however, the previoulsy observed SF at $x = 5.5\,\mathrm{m}$ did not develop and the supershear transition took place. This is confirmed by the deformation and strain fields measured in the close-up and full-view perspectives. The latter additionally revealed that the super critical crack length was around $x = 5.5\,\mathrm{m}$.

Until $x = 5\,\mathrm{m}$, crack propagation speeds in all three PSTs were very similar and dominated by mixed-mode anti-crack propagation. The deformation fields revealed bending of the slab and measured crack propagation speeds were sub-Rayleigh (Extended Data Fig. 5).

Supplementary Note 2 – Strain differences between close-ups

In the main manuscript, the crack driving strain localization measured in the close-ups is illustrated in Figure 3. If the crack propagates as a mixed-mode anti-crack the normal strain in the weak layer dominates (Figure 3C). After the transition to the supershear crack propagation regime, the cracking is due to shear strain in the weak layer (Fig. 3E). The respective other fields (Extended Data Figure 4), revealed no pronounced strain localization in the close-up measurements. For cracking in the mixed-mode anticrack regime, there is no shear strain localization at the crack tip (Extended Data Figure 4C, $x \approx 2.35\,\mathrm{m}$). For cracking in the supershear propagation regime, there is no pronounced localization of normal strain around the crack tip (Extended Data Figure 4E, $x \approx 9\,\mathrm{m}$).

Supplementary Note 3 – MPM simulations of crack propagation with a layered slab

We performed two-dimensional Material-Point-Method (MPM) simulations of 80 to 150 m long PST experiments to evaluate potential changes in crack speed for multi-layered snow slabs. More precisely,

we analyzed whether the approximation of a layered slab as an equivalent homogeneous slab with a mean density and elastic modulus is appropriate to evaluate the mean shear wave speed and identify the supershear regime.

The MPM, mechanical models for the slab and weak layer and crack tip identification technique used here can be found in (14) and (11). The parameters for these simulations can be found in Extended Data Table 2. A slight variation of the Cohesive Cam Clay model presented in (14) was used in these simulations. For simplicity, the softening law was replaced by an instantaneous and complete shrinkage of the yield surface (equivalent to $\alpha = +\infty$).

Three configurations of density layered slab of equal mean density were considered: homogeneous (h), increasing (a) and decreasing (b) density with distance to the buried weak layer. The density values used were $\rho^- = 2\rho_m/3$, ρ_m and $\rho^+ = 4\rho_m/3$ for the low, medium and high density, respectively, with $\rho_m = 100\ kg.m^{-3}$ the mean density. The Young's modulus of each layer was then computed using Sigrist's empirical parameterization (see section on elastic waves) (9) to evaluate $c_e^H = \sqrt{E^{sig}(\rho_m)/\rho_m}$, $c_e^+ = \sqrt{E^{sig}(\rho^+)/\rho^+}$ and $c_e^- = \sqrt{E^{sig}(\rho^-)/\rho^-}$. The results of these simulations are shown in Extended Data Figure 7.

The homogeneous (h) configuration is a reference for which we know the behavior and the supershear nature of the crack propagation (11)). Configuration (b) is an approximation of the field experiments presented in this paper. Its steady state-crack speed differs by only 4.8% of that of the homogeneous slab. Thus, while not negligible, the equivalent homogeneous slab approximation appears appropriate. Importantly, we verified here that the crack speed is not only driven by the modulus of the stiffest layer.

In addition, the shear strain rate in the supershear regime exhibits a so-called supershear Mach cone as well as a dilatation (pressure) lobe initiated at the crack tip. These features are characteristic of a supershear (also called intersonic) regime (15; 16). However, this behavior was not visible in the field experiment for the following reasons. First, the distance between supershear transition ($5.5\,\mathrm{m}$) and measurement (close-up in PST #3) was too close. As MPM simulations suggest, supershear cracking was not yet in a steady-state. In a transitional regime, strain localisation of a Mach cone cannot be expected to show clear signatures. Second, the FOV of the close-up in PST #3 was too focused on the crack tip. To discriminate the supershear Mach cone from the dilatation lobe (Figure 9) a larger field of view, containing the whole slab, would be necessary. Thus, in order to capture a supershear Mach cone in experiments, one would need even longer columns, a FOV containing the full height of the slab and a measurement resolution comparable or better than in our close-ups.

Supplementary Table 3: MPM simulation parameters used to produce Figure 9.

MPM Simulation Parameters		Slab	Weak Layer
Geometry	Thickness (m)	0.8	0.05
	Length (m)	150	150
	Slope angle (°)	37	37
Material properties	Poisson's ratio ν	0.2	0.2
	Density[1] ρ (kg m^{-3})	67 - 133	100
	Young's modulus[2] E (MPa)	0.44 - 3.34	2.0
	Initial consolidation pressure p_0 (kPa)	-	25
	Tension/compression ratio β	-	0.35
	Friction coefficient M	-	1.3
	Hardening factor ξ	-	0.01
Numerical model	Element size[3] dx (cm)	0.5 - 1.5	
	Mean number of particles per cell	4	
	CFL	0.6	
	Frame rate (s^{-1})	1000	

[1] ρ values include 67, 100 and 133

[2] E values include 0.44, 1.43 and 3.34

[3] values include 0.5 (f. and g.), 1.0 (d. and e.) and 1.5 (a., b. and c.)

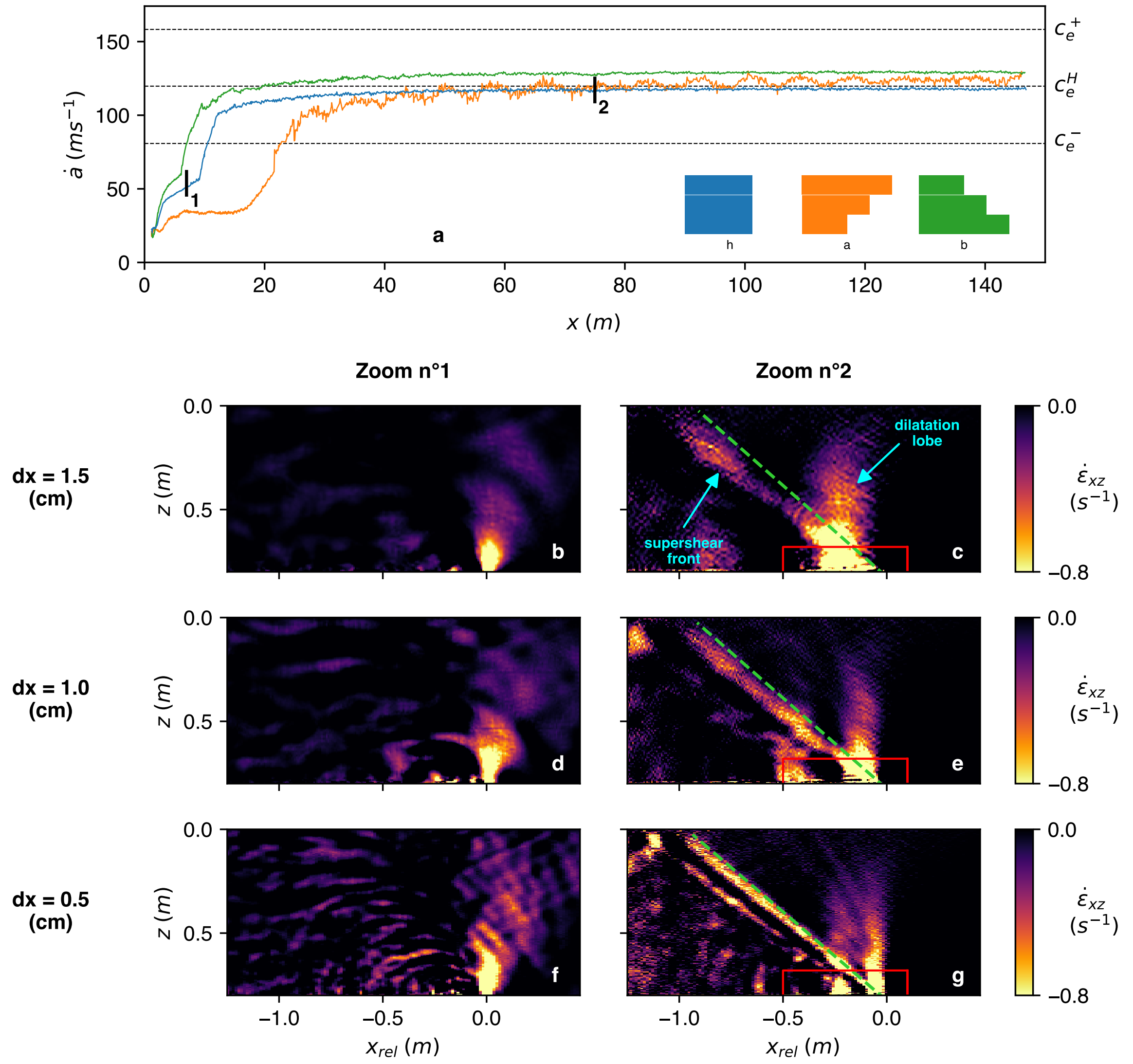

Supplementary Figure 9: 2D MPM simulation of long PST experiments with layered snow slabs. **a**, Crack speed in a 150 m long PST for three different slab layering (each has a mean density of $\rho_m = 100\ kg\ m^{-3}$ and a cell size of dx = 1.5 cm). c_e^H, c_e^- and c_e^+ are the expected supershear crack speeds (11) for a homogeneous slab of density ρ_m, ρ^- and ρ^+, respectively. The steady-state crack speed is derived by taking the average crack speed after $100\,\mathrm{m}$ of crack propagation distance. Profile h corresponds to the homogeneous case; profile a corresponds to an heterogeneous slab with density decreasing with increasing depth; profile b corresponds to an heterogeneous slab with density increasing with increasing depth. **b** to **g** are zoomed plots of the shear rate of deformation in the homogeneous (h) slab in the anticrack (**b**, **d** and **f**, Position 1 in **a**) and supershear regime (**c**, **e** and **g**, Position 2 in **a**) for different resolutions. Positions 1 and 2 in A are at (7.00 ± 0.04) m and (75.0 ± 0.1) m, respectively. $x_{rel} = 0$ m corresponds to the position of the crack tip. On **c**, **e** and **g**, the theoretical supershear Mach cone, with an angle of $\beta = \arcsin(c_S/V)$ where V is the mean crack speed between 65 and 75 m, is represented by the green dotted line. The red contour shows the FOV of the close-up measured in field experiment PST #3.

Supplementary Note 4 – Calculation of the supercritical crack length based on Trottet et al.

Let us recall the formula for the supercritical crack length from Trottet et al. (11):

$$a_{sc} = \Lambda \frac{\tau_p - \tau_g}{\tau_g - \tau_r}$$

where

$$\Lambda = \sqrt{\frac{E' D D_{wl}}{G_{wl}}}$$

with E' being the plane strain elastic modulus, D and D_{wl} representing the slab and weak layer thicknesses, respectively, and G_{wl} the shear modulus of the weak layer. Additionally, the shear stress induced by the slab load is given by

$$\tau_g = \rho g D \sin\psi,$$

where ρ is the density of the material, g the gravitational acceleration, and ψ the slope angle. The residual frictional resistance is represented by

$$\tau_r = \mu_d \rho g D \cos\psi,$$

where μ_d is the dynamic (or crack face) friction coefficient. The shear strength of the weak layer is given by

$$\tau_p = c + \mu\sigma_n,$$

where μ is the internal friction coefficient, σ_n is the normal stress, and c is the cohesion of the weak layer. The cohesion of the weak layer can thus be calculated using the following equation:

$$c = \frac{a_{sc}}{\Lambda}\left(\tau_g - \tau_r\right) + \tau_g - \mu\sigma_n$$

This calculation is particularly useful as it allows for a direct comparison between values obtained through back-calculation and shear frame measurement data from the literature. Assuming $\mu_d = 0.6$ (17), $\mu = 0.36$ (18), $F_{wl} = \frac{D_{wl}}{G_{wl}} = 10^{-7}$ m/Pa (19), using the elastic modulus evaluation from (9) and assuming $a_{sc} = 5.5$ m from the experiments (PST 3), we obtain a cohesion value of $c = 1.4$ kPa, which falls within the range of values measured by (20) and (21). Conversely, performing the inverse calculation by assuming a realistic range of cohesion values between 0.5 and 2 kPa yields a range of supercritical crack lengths between 1.25 and 7.3 m. While this analysis does not provide a single definitive prediction due to uncertainties in snow property measurements, it strengthens the conclusions of our study regarding the Burridge-Andrews mechanism (see Discussion Section in main text). This analysis is further reinforced by the numerical reproduction and analysis of PST 3 on the basis of MPM and DEM simulations (see main text and Supplementary Note 5).

Supplementary Note 5 – Numerical reproduction of the supershear transition in PST 3 using the Discrete Element Method.

We used the 3D DEM model developed to investigate crack propagation behavior in PST experiments (22; 23). Initially introduced by Cundall and Strack (1979) (24), DEM is a numerical tool that employs numerous discrete interacting particles and is commonly used to study large deformations in granular-like assemblies. Here, we use the commercial software PFC3D (v5), developed by Itasca. The simulated PSTs consist of three layers: a rigid basal layer, a transversely isotropic weak layer similar to layers of surface hoar or facets and a dense, uniform slab layer. The weak layer was created using cohesive ballistic deposition, resulting in a porosity of 80% and a thickness (D_{wl}) of 0.02 m. The slab layer was generated using cohesionless ballistic deposition, with a porosity of 45% and a thickness (D) of 0.8 m. We employed the parallel-bond contact model (PBM) for each layer to simulate particle interactions. The slab is isotropic and has a macroscopic density of $\rho = 102\ kg.m^{-3}$ based on the measured mean density in the experiment. Using the fit presented by Bobillier et al. (25), we adjusted the slab bond elastic modulus to match the estimated slab shear wave speed. For the weak layer properties, we used the parameters published in Bobillier et al. (23) and adjusted the weak layer strength to reproduce the measured critical crack length. The slope angle was set to 37°. The DEM PST model, with a length of 30 m and a data acquisition time of 2 ms, requires approximately one week of computing time on a high-performance workstation. Figure 10 shows the results of the simulation which very closely match the experimental results as well as the results from MPM simulations presented in the main text.

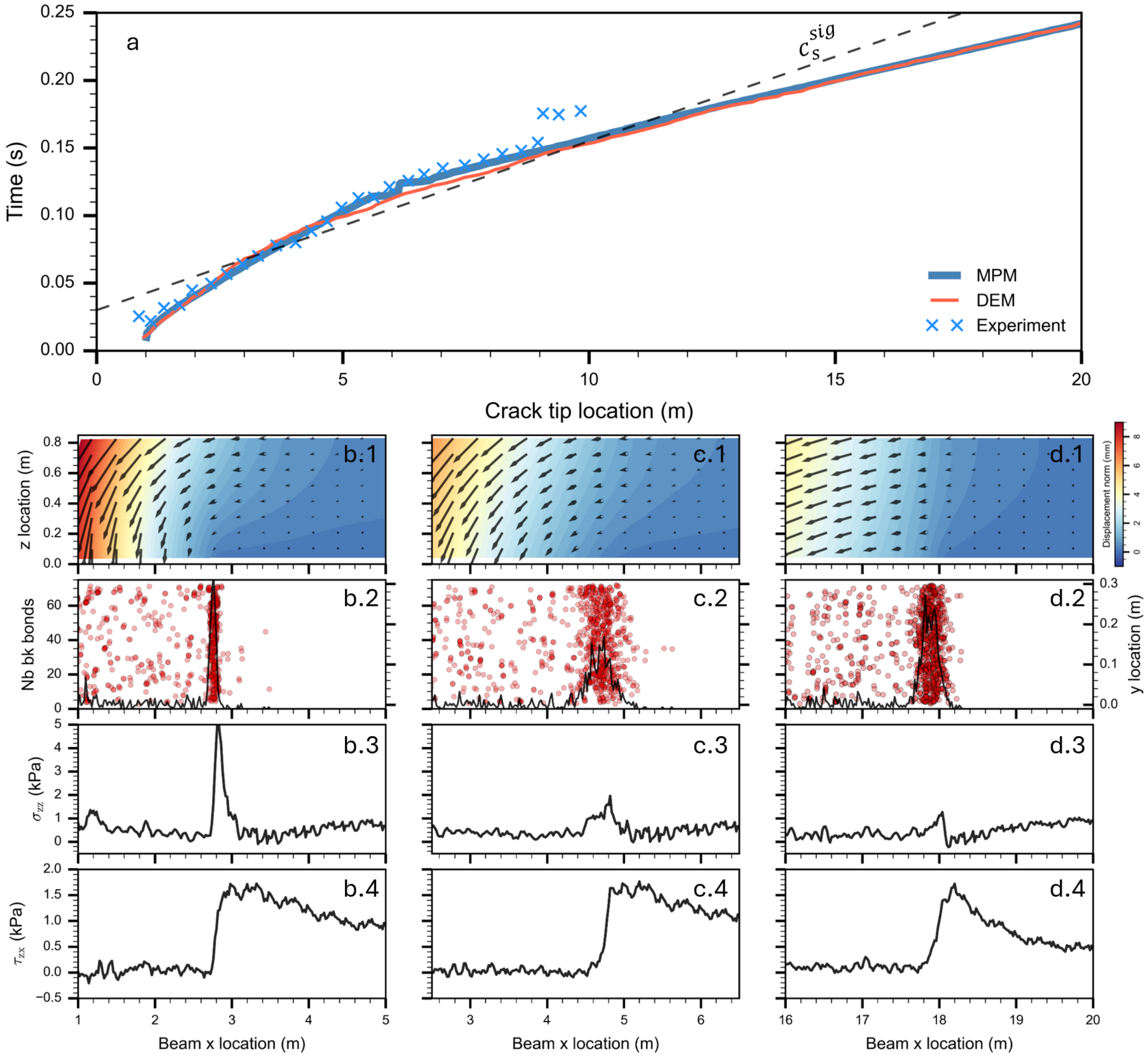

Supplementary Figure 10: Crack propagation dynamics and stress state obtained from DEM-PST simulations. (a) Spatio-temporal evolution of the crack tip. The orange line corresponds to the DEM simulation, the blue line to the MPM simulation, and the blue dots to experimental data. The dashed black line represents the crack tip location corresponding to the slab shear wave speed. (b-d) show the crack state for three crack tip locations: 2.1 m, 5 m, and 18 m. (1) Slab displacement magnitude. (2) Top view of the weak layer showing bond states, with red indicating bonds breaking at the current time step. The black line represents the breaking bond distribution along the beam's length. (3) Normal stress σ_{zz} and (4) shear stress τ_{zx}. DEM results closely match MPM in terms of crack propagation speed in both sub-Rayleigh and supershear regimes, as well as in displacement and stress distribution. Additionally, at the supershear transition, we observe the nucleation of a daughter crack ahead of the main crack, evidenced by a two-peak bond-breaking distribution—similar to MPM. Due to the discrete nature of the simulation and microscale heterogeneity, the crack appears slightly more diffuse than in the continuum-based, homogeneous MPM simulation.

Supplementary References

[1] C. Fierz, *et al.*, *UNESCO-International Hydrological Program. (Technical Documents in Hydrology)* **83**, 90 pp (2009).

[2] C. K. Chui, H. Mhaskar, *Applied and Computational Harmonic Analysis* **28**, 104 (2010).

[3] S. B. Damelin, N. Hoang, *International Journal of Mathematics and Mathematical Sciences* **2018**, 1 (2018).

[4] A. Buades, B. Coll, J. Morel, *IEEE Conference on Advanced Video and Signal Based Surveillance, 2005.* (2005), pp. 70–74.

[5] C. Scapozza, F. Bucher, P. Amann, W. J. Ammann, P. Bartelt, *Annals of Glaciology* **38**, 291 (2004).

[6] B. Bergfeld, *et al.*, *Nat. Hazards Earth Syst. Sci.* **23**, 293 (2023).

[7] B. Gerling, H. Löwe, A. van Herwijnen, *Geophys. Res. Lett.* **44**, 11 (2017).

[8] A. van Herwijnen, *et al.*, *Journal of Glaciology* **62**, 997 (2016).

[9] C. Sigrist, Measurements of fracture mechanical properties of snow and application to dry snow slab avalanche release, Ph.D. thesis, ETH Zürich (2006).

[10] J. Gaume, A. van Herwijnen, G. Chambon, K. Birkeland, J. Schweizer, *The Cryosphere* **9**, 1915 (2015).

[11] B. Trottet, *et al.*, *Nature Physics* **18**, 1094 (2022).

[12] D. Szabo, M. Schneebeli, *Applied Physics Letters* **90**, 151916 (2007).

[13] A. van Herwijnen, *Canadian Geotechnical Journal* **50**, 1044 (2013).

[14] J. Gaume, T. Gast, J. Teran, A. van Herwijnen, C. Jiang, *Nature Communications* **9**, 3047 (2018).

[15] V. Rubino, A. Rosakis, N. Lapusta, *Journal of Geophysical Research: Solid Earth* **125**, e2019JB018922 (2020).

[16] M. Gori, V. Rubino, A. Rosakis, N. Lapusta, *Nature Communications* **9**, 4754 (2018).

[17] A. van Herwijnen, J. Heierli, *Geophys. Res. Lett.* **36** (2009). L23502.

[18] I. Reiweger, J. Gaume, J. Schweizer, *Geophys. Res. Lett.* **42**, 1427 (2015).

[19] B. Richter, J. Schweizer, M. Rotach, A. van Herwijnen, *The Cryosphere Discussions* pp. 1–21 (2019).

[20] J. Jamieson, C. Johnston, *Ann. Glaciol.* **32**, 59 (2001).

[21] B. Jamieson, J. Schweizer, *J. Glaciol.* **46**, 151 (2000).

[22] G. Bobillier, *et al.*, *Scientific Reports* **11**, 11711 (2021).

[23] G. Bobillier, *et al.*, *Natural Hazards and Earth System Sciences Discussion* pp. 1–15 (2024).

[24] P. A. Cundall, *Ingenieur-archiv* **59**, 148 (1989).

[25] G. Bobillier, *et al.*, *Granular Matter* **26**, 1 (2024).
