# Supplementary material for: Signatures of the sub-Rayleigh to supershear fracture transition in snow avalanche experiments
Source: Nat Commun. 2025 Dec 16;16:11153. doi: 10.1038/s41467-025-65825-6 (PMC12708799; doi:10.1038/s41467-025-65825-6)
Supplement: Supplementary file 2 — Description of Additional Supplementary Information [file 41467_2025_65825_MOESM2_ESM.pdf]

Description of Additional Supplementary Files for

Signatures of the sub-Rayleigh to supershear fracture transition in snow avalanche experiments

Bergfeld et al.

To whom correspondence should be addressed; E-mail: johan.gaume@slf.ch

Captions for Movies SM1 to SM7

Supplementary Movie SM1

Video of the experimental avalanche consisting of a snow fracture test performed in Davos, Switzerland.

Supplementary Movie SM2

Spatio-temporal patterns of displacement vectors (top), normal and tangential displacements, normal and shear strain at the close-up of PST #2.

Supplementary Movie SM3

Spatio-temporal patterns of displacement vectors (top), normal and tangential displacements, normal and shear strain at the close-up of PST #3.

Supplementary Movie SM4

Temporal evolution of crack propagation speed (top) and shear strain rate, revealing a Mach cone during steady state supershear crack propagation.

Supplementary Movie SM5

Spatio-temporal patterns of displacement vectors in PST #1. The vectors are colored by their orientation.

Supplementary Movie SM6

Spatio-temporal patterns of displacement vectors in PST #2. The vectors are colored by their orientation.

Supplementary Movie SM7

Spatio-temporal patterns of displacement vectors in PST #3. The vectors are colored by their orientation.
